# Styles, strategies, and challenges of PA leaders nationally: An exploratory qualitative study

**DOI:** 10.1097/01.JAA.0000000000000140

**Published:** 2024-10-29

**Authors:** Bethany Grubb

**Affiliations:** **Bethany Grubb** is an assistant professor in the PA program at the UT Southwestern School of Health Professions in Dallas, Tex. The study was conducted during the author's American Academy of Physician Associates-PA Education Association 2022-23 research fellowship. The author has disclosed no potential conflicts of interest, financial or otherwise.

**Keywords:** leadership, teams, psychologic safety, collaborative, emotional intelligence, constructive feedback

## Abstract

**Objectives:**

To identify the leadership styles, strengths, strategies, and key factors of PA leaders in healthcare executive, clinical, and academic settings.

**Methods:**

An exploratory, qualitative study was completed through the American Academy of Physician Associates 2023 Practice Survey to answer seven qualitative questions. Of the 1,423 PAs who responded to the survey invitation, 348 PA leaders in formal and informal roles chose to answer, for a 24.4% response rate.

**Results:**

For PA leaders' styles and strengths, collaborative and emotional intelligence emerged as themes. For key factors of PA leadership, balancing expectations and giving and receiving critical feedback emerged as themes. For skills or qualities PA leaders wish they had before starting to lead, project management skills and increased confidence emerged as themes.

**Conclusions:**

Leadership development training needs to be in the didactic and clinical curriculum of PA programs for the future of PA leadership. Leadership pathways for PA leaders in healthcare executive, clinical, and academic settings need to be created and established more widely.

Formal leadership training or curriculum rarely is offered nationally in medical schools or physician associate/assistant (PA) programs. Few leadership pathways exist for PAs in healthcare executive, clinical, and academic roles, but organizational opportunities are growing. In clinical roles, PAs lead their teams as chief advanced practice provider (APP) officer, lead APP, or inpatient medical director.^1^ The population of PAs holding a clinical leadership role nationally has yet to be discovered. In academic roles, PAs serve as dean/associate dean, department chair/head/division chief, and program director.^2^ In healthcare executive roles, PAs serve as directors of PAs and NPs, system directors of PAs and NPs, or chiefs of advanced practice. In 2017, Bushardt and colleagues determined that 500 to 3,600 PAs were in healthcare administration, although this likely is an underestimation in 2024.^3^ Two areas of leadership literacy are important: interpersonal and healthcare systems literacy. Coordinating teams, coaching and giving feedback, facilitating interprofessional communication, and displaying emotional intelligence are all key skills to effective leadership. The American Medical Association, the United Kingdom's National Health Service, and the Canadian College of Health Leaders all recognize these skills as core competencies for leaders. The importance of creating leadership development training for PAs and NPs to successfully transition into leadership roles cannot be stressed enough.^4^-^6^

Healthcare delivery typically occurs via teams.^7^,^8^ PAs, NPs, and physicians participate as members and may take on team leadership responsibilities; sometimes, this leadership is shared. Teamwork's effect on delivering patient care and healthcare outcomes is crucial going forward. The leadership of any team is critical to managing conflict and shaping team culture and norms.^8^ The best practices of leading teams are not well known because most studies focus on specific tasks or goals of leadership rather than the psychosocial components of leadership, including creating psychologic safety, building trust, and effectively using mental models in healthcare delivery. Future research examining team member roles, effective leadership, and collaboration outcomes is critical.

This study sought to better understand PA leaders and their leadership in healthcare executive, clinical, and academic roles. Leadership on teams is challenging because of the complexities of balancing multiple expectations of team members and organizational leadership. Gaining a more robust understanding of the challenges faced by PA leaders and the strategies to overcome these challenges can help the profession better equip future PA leaders.

## METHODS

The study was approved by the UT Southwestern institutional review board. The following research questions were addressed:

- Research question 1 (RQ1): What are the common leadership styles, strengths, and strategies in PA leadership?
- Research question 2 (RQ2): What are the key factors affecting PA leadership?
- Research question 3 (RQ3): What do PAs in leadership wish they had known before they started leading?

An exploratory, qualitative study was completed through the American Academy of Physician Associates (AAPA) 2023 Practice Survey available from March to May 2023. The survey was sent to about 30,000 PAs with valid email addresses who had not opted out of research surveys. A total of 11,995 emails were delivered, and 1,423 PAs responded to the invitation, for a response rate of 11.9%. After completing the survey, those who identified as a formal leader or as being in an informal leadership role could opt to respond to questions about leadership in the same sitting. After providing informed consent, 348 participants answered a series of questions about leadership (response rate 24.5%). Respondents could answer seven qualitative questions about leadership style, strengths, and strategies. The questions were:

- How would you describe your leadership style?
- What are your strengths in leadership?
- What strategies do you use to incorporate your strengths into your leadership role?
- What are the challenges facing PAs in leadership?
- What are the strategies that you use to overcome the challenges faced?
- What qualities do PAs in leadership perceive are lacking in their employees?
- What do PAs in leadership wish they had known before they started leading?

Codes and themes emerged from the data using Dedoose software. In qualitative studies, coding to saturation is an established best practice of data analysis, and this was followed. Code saturation is based on the frequency of codes or themes in the data analysis process, generally meaning that no new codes or themes have emerged. At saturation, the codes that emerge overlap with already established codes. An analysis was conducted to test interrater reliability, and any differences in coding were discussed and consensus was reached.

## RESULTS

Of the 348 respondents, those between ages 40 and 54 years had the highest percentage of formal leadership roles (63% to 67%) compared with informal roles (32% to 35%). Respondents ages 65 years and older had the lowest percentage of formal leadership roles (38%), but the highest percentage of informal leadership roles (62%). Respondents under age 30 years were more likely to hold an informal leadership role (53%) than a formal role (45%). The distribution of formal leadership roles (58%) compared with informal roles (41%) was equal between male and female PA leaders. Sixty-eight percent of respondents in a formal leadership role held a doctoral degree. More than 60% of PA leaders identifying as White were in a formal leadership role, compared with 38% and 30%, respectively, for PAs identifying as Black and PAs identifying as Asian American or Pacific Islander. Only the top five themes for each qualitative question are discussed, so the percentages do not equal 100 because themes with lower percentages are not included.

### Leadership styles strategies

RQ1 asked: *What are the common styles and strategies in leadership among PA leaders?* Participants were asked to describe their leadership style, their strengths in leadership, and strategies they used to incorporate their strengths into their leadership roles.

- *Leadership style*. The five major themes that emerged from the coded data were collaborative/democratic (36%), lead by example (29%), servant (13%), transformational (9%), and coaching (4.8%). PAs who lead with the collaborative/democratic style said, “collaboratively work with others,” “supportive, providing positive feedback,” “proactive and encourage others to do things in the way that works for them, then we get together and review,” “collaborative, fair, equitable,” and “team approach.” Leading by example was the second most common style of leadership to emerge from participant responses. PAs who lead with this style said, “strong and relatable, don't ask others to do anything I wouldn't do myself,” “my actions and work ethic show my leadership style,” “demonstrating the qualities that I think are important,” and “modeling.”
- *Strengths in leadership*. The five major themes that emerged from the coded data were listening (26%), motivating others (24%), mission focus (12%), transparency (9%), and process improvement (7%). PA leaders whose greatest strength was listening said, “listening and encouraging,” “calm demeanor, approachable, good communicator,” and “able to listen.” PA leaders who motivate others said, “to motivate others to want to be greater,” and “I have high EQ [emotional intelligence] and use it to find out what motivates people and what is frustrating them.”
- *Strategies to incorporate strengths into the leadership role*. The five major themes that emerged from the coded data were clear communication (28%), psychologic safety (27%), setting goals and expectations (21%), organization (8%), and debriefing teams (8%). PA leaders who use clear communication as a strategy said, “open sessions with multiple managers to discuss shared problems,” “clear communication and feedback (both ways),” “open communication,” “meet regularly, provide timely guidance,” and “repeat things in different ways.” PA leaders who create psychologic safety said, “mutual respect, avoidance of top-down leadership,” “walk around and find common ground and/or differences in experiences,” and “curiosity, genuine listening, holding each team member in genuine, unconditional positive regard.”

### Factors affecting PA leadership

RQ2 asked: *What are the key factors affecting PA leadership?* Participants were asked to describe the leadership challenges and the strategies used to overcome these challenges in their leadership roles.

- *Challenges facing PA leaders*. The five major themes that emerged from the coded data were conflict avoidance (22%), setting boundaries (20%), criticism (19%), lack of confidence (12%), and not delegating (11%). PA leaders who find conflict a challenge said, “engaging people who want to be part of a project but do not have the appropriate skills but think they do,” “confrontation,” “don't like confrontation,” “confronting people regarding difficult issues,” and “averse to conflict.” The second most common challenge was setting boundaries. PA leaders who find setting boundaries a challenge said, “I can sometimes be too hands-on,” “work/life balance and time,” “knowing when to say no, often overextend myself for others,” and “overworking.”
- *Strategies used to overcome the challenges faced*. The five major themes that emerged from the coded data were giving and receiving positive and negative feedback (26%), self-reflection (15%), accountability (14%), persistence (14%), and resetting the frame of reference (14%). PA leaders who use giving and receiving positive and negative feedback as a strategy to overcome challenges said, “meet with others 1:1 in private to correct and coach,” “seek out feedback from my direct reports and administrative leaders” “carefully word what I need to stay in a way that is not aggressive or off-putting,” “positive reinforcement on the floor, if negative feedback needed that is done one-on-one behind closed doors,” and “ask open-ended questions, seek input and feedback from my team.” The second most common strategy to overcoming challenges was self-reflection. PA leaders who use self-reflection said, “take the time to reassess challenges, look for ways I could have handled it for a different outcome,” “calming techniques,” “take a step back,” and “thoughtful reflection.”
- *Qualities PAs in leadership perceive as lacking in their employees*. The five major themes that emerged from the coded data were initiative (36%), altruism (14%), communication (11%), integrity (11%), and critical thinking/problem-solving (8%). PA leaders who perceived a lack of initiative in their employees said, “desire to be involved,” “self motivation,” “work ethic,” “ability to self-direct and work hard on their own,” “taking initiative,” “lack of motivation to take on additional responsibilities, foster their potential and function outside their comfort zone,” and “asking for assistance.” The second most common quality that PA leaders perceived as lacking in employees was altruism. PA leaders who perceived a lack of altruism in employees said, “motivation to create change,” “desire to do more than the bare minimum,” “team building,” “commitment to the company,” and “desire to go above and beyond the bare minimum of duties to improve the department as a whole.”

### What leaders wish they had known

RQ3 asked: *What do PAs in leadership wish they had known before they started leading?* The five major themes that emerged from the coded data were project management (32%), increased confidence (12%), accepting people where they are (11%), navigating toxic work environments (11%), and the importance of social capital (9%). PA leaders who wished they had come into leadership with project management skills said, “how to perform interviews and onboarding,” “transition of priorities from patient care to administration,” “how hospital systems are set up, a better understanding of how billing works in a complex hospital setting,” “team building instead of doing everything myself,” “better organization,” “levels of management and how to navigate,” and “set priorities.” The second most common response was increased confidence. PA leaders who wished they had more confidence said, “the importance of how much you can change just by having a voice,” “salary negotiation,” “can't make everyone happy,” “public speaking,” and “fear less, trust myself more.”

## DISCUSSION

### RQ1: Common leadership styles, strengths, and strategies

The first theme was collaboration and emotional intelligence. A collaborative or democratic leadership style emerged as a distinct theme for PA leadership and in evaluating the strengths and strategies of PA leaders. Of Goleman's six leadership styles, democratic leadership welcomes new ideas from all team members and strives for a consensus in decision-making processes.^9^ A democratic leadership style empowers the team but typically takes time, so it may not be the best style when fast decisions need to be made.

One of the most important ways to facilitate collaboration is through clear communication and creating psychologic safety with colleagues, employees, and stakeholders. In the qualitative question about leadership strategies, most participants reported clear communication and psychologic safety as critical factors in team leadership. Psychologic safety lets team members ask questions, admit mistakes, or offer a dissenting opinion without the risk of penalty.^10^ Psychologic safety in teams is one of the strongest predictors of high team performance.^10^

Emotional intelligence emerged as a distinct leadership style, strength, and strategy theme. Goleman's five characteristics of emotional intelligence are self-awareness, self-regulation, motivation, empathy, and social skills.^9^ Emotional intelligence encompasses PA leadership's two highest strengths, listening and motivating others. Listening to and motivating others requires emotional intelligence.

### RQ2: Key factors in leadership: equipoise and giving and receiving critical feedback

Equipoise, or the balancing of forces or interests, emerged as a distinct theme for challenges PAs face in leadership. The question asked what challenges PAs face as leaders and 42% of participants said avoiding conflict and setting boundaries. Conflict often arises because of competing interests, goals, or expectations, and one of the greatest challenges of leading is to balance all of these factors. Setting boundaries is one way to create balance when leading, but is one of the hardest decisions for many. At times, team members' lack of initiative or altruism also pose challenges to leading teams effectively. When team members lack motivation or the desire to go above and beyond, leaders risk expending high amounts of energy on individual team members and may lose vision.

Giving and receiving critical feedback emerged as a distinct theme in how PA leaders overcome challenges faced. Effective healthcare teams share six team characteristics: purpose, goals, leadership, communication, cohesion, and mutual respect.^11^ Leadership emphasized the importance of directing activities toward the team's goals. Effective leaders managed conflict, made decisions, shared ideas, maintained open lines of communication, coordinated tasks equally, provided constructive feedback, supported team members, and maintained the team's trust. Shared leadership of the team also was an important component. These team characteristics are highly influenced by the team leader and the culture created within the team to facilitate communication, create cohesion, and maintain mutual respect for one another, even with differing ideas and opinions.

### RQ3: Skills or qualities PAs in leadership wished they had before becoming leaders

The leading themes raised by this question were project management and increased confidence. PAs often find themselves in leadership positions because they are astute clinicians but often lack formal leadership development training before they assume leadership roles. Leading a team of people requires a different skill set, including resolving conflicts, meeting quality metrics, and balancing expectations of organizational leadership. PA leaders identified specific skills under the umbrella of project management that they wished they had before leading, specifically identifying onboarding, billing within a complex hospital system, setting priorities, using team members effectively, and managing expectations of organizational leadership as areas in which they wished they had prior experience.

PAs in leadership also wished they had increased confidence, which is needed for negotiation situations, especially with salaries; speaking publicly; using one's voice for change; and acknowledging that not everyone will be happy with decisions.

Nursing, midwifery, and allied healthcare leaders have identified qualities of transformational leadership to strengthen healthcare leadership. Most of the literature found was from the United Kingdom and Australia.^12^,^13^ The most important skills identified were critical thinking, systems thinking, collaboration, responsiveness to diverse needs of multiple stakeholders, and the ability to persuade and encourage adoption of change.^14^,^15^ With the majority of the literature on leadership originating in Australia and the United Kingdom, there is ample opportunity for PAs to contribute to this body of knowledge in the United States.

## LIMITATIONS

The survey delivery was more typical of surveys gathering quantitative data, although qualitative data were acquired. The opportunity to use purposive sampling, which is more common with a qualitative approach, was not feasible. The survey response rate for the AAPA Practice Survey was relatively low (11.9%), which can lead to nonresponse bias, but was comparable to previous years. If the sample does not accurately represent the PA leadership population, then the conclusions for PA leadership may be limited. Certain groups of PA leaders may be over- or underrepresented, affecting validity and reliability.

## CONCLUSIONS

To improve PA leadership skills in the future, leadership development training needs to be part of the didactic and clinical curriculum of PA programs. Leadership pathways for PA leaders in healthcare executive, clinical, and academic roles need to be created and established. Healthcare delivery is happening on teams, which allows PAs to lead in healthcare executive, clinical, and academic settings. Creating psychologic safety, giving and receiving constructive feedback, and project management will be critical skills for PA leaders and aspiring PA leaders as leadership opportunities continue to grow. More research is necessary to validate the study's findings, perhaps through in-depth interviews or a qualitative approach called phenomenology. Additional quantitative research could further identify transformational leadership styles. Pathways to leadership for PAs are not clearly defined, unlike in nursing, where leaders can serve as director of nursing or chief nursing officer. The leadership pathways of those who are in leadership roles, whether healthcare executive, clinical, or academic, are important to define as we consider the expansion of leadership for PAs. Creating leadership development training, whether at the organizational level or in PA programs, is important to further determine policy recommendations for PA educators and healthcare systems.
